# Endowing Orthopedic Implants’ Antibacterial, Antioxidation, and Osteogenesis Properties Through a Composite Coating of Nano-Hydroxyapatite, Tannic Acid, and Lysozyme

**DOI:** 10.3389/fbioe.2021.718255

**Published:** 2021-07-19

**Authors:** Guofeng Wang, Yaxin Zhu, Xingjie Zan, Meng Li

**Affiliations:** ^1^The Fourth Affiliated Hospital of China Medical University, Shenyang, China; ^2^Oujiang Laboratory, Wenzhou Institute, University of Chinese Academy of Sciences, Wenzhou, China

**Keywords:** hydroxyapatite, polyphenol, orthopedic coatings, multi-functionality, osteogenesis

## Abstract

There is a substantial global market for orthopedic implants, but these implants still face the problem of a high failure rate in the short and long term after implantation due to the complex physiological conditions in the body. The use of multifunctional coatings on orthopedic implants has been proposed as an effective way to overcome a range of difficulties. Here, a multifunctional (TA@HA/Lys)_n_ coating composed of tannic acid (TA), hydroxyapatite (HA), and lysozyme (Lys) was fabricated in a layer-by-layer (LBL) manner, where TA deposited onto HA firmly stuck Lys and HA together. The deposition of TA onto HA, the growth of (TA@HA/Lys)_n_, and multiple related biofunctionalities were thoroughly investigated. Our data demonstrated that such a hybrid coating displayed antibacterial and antioxidant effects, and also facilitated the rapid attachment of cells [both mouse embryo osteoblast precursor cells (MC3T3-E1) and dental pulp stem cells (DPSCs)] in the early stage and their proliferation over a long period. This accelerated osteogenesis *in vitro* and promoted bone formation *in vivo*. We believe that our findings and the developed strategy here could pave the way for multifunctional coatings not only on orthopedic implants, but also for additional applications in catalysts, sensors, tissue engineering, etc.

## Introduction

The global sale of orthopedic implants was about $36.5 billion in 2017 and is expected to reach $47.1 billion in 2024, as forecasted by Evaluate Med. Tech. (Moore et al., 2018). However, the current clinical failure rate of such implants is unacceptable (10–20% at 5–10 years after implantation, based on the data from multiple centers) (Schroer et al., 2013), which affects the daily life of millions of patients annually and places a huge burden on public health systems. Improving the failure rate is thus a major focus worldwide.

Aseptic loosening originating from the generation of wear particles and micromotion of the implant, leading to chronic inflammation and poor osseointegration (Sundfeldt et al., 2006), as well as the infection caused by microbes and/or bacteria during and/or after surgery, are the two major factors associated with implant failure (Campoccia et al., 2006). To address these issues, dual-function implants with enhanced osseointegration and strong antibacterial abilities have been proposed and extensively explored in recent years (Min et al., 2016; Raphel et al., 2016). Direct incorporation of antibacterial and osteogenic elements into metal implants is straightforward (Wang et al., 2017), but limited by the changes of mechanical properties, long-term stability in the body, the controllable release of incorporated drugs, etc. The implant needs to support cell attachment, proliferation, and differentiation, to fulfill the goal of filling the gap between bone tissue and implant. Considering that cellular events and bacterial colonization occur on the surface of implants, in recent years there have been numerous investigations of engineering implant surfaces with enhanced osseointegration and strong antibacterial abilities, through roughening and/or patterning the surface (Boyan et al., 1996), attaching bioactive molecules (peptides, proteins, RNA, etc.) (Rammelt et al., 2004; Elmengaard et al., 2005; Fröhlich, 2019), and incorporating bactericidal constituents (inorganic particles, antibiotics, etc.) (Antoci et al., 2008; Shuai et al., 2018). However, there is an urgent need to resolve issues such as antibiotic resistance caused by the misuse of antibiotics (Stewart and William Costerton, 2001), cellular toxicity induced by inorganic particles (Soenen et al., 2011), and the deficiency of attached bioactive molecules due to low density and/or deactivation in order for the process of clinical translation to progress.

The materials applied on implants should be non-immunogenic and biocompatible with the host tissue. Besides, the ability to osteogenesis was one of the most significant profiles should be considered. Various materials, including a metal alloy, calcium phosphate, a bioactive glass, etc. have been widely used for fabricating implants or implant coatings by diverse techniques (Surmenev and Surmeneva, 2019). Hydroxyapatite (HA), an inorganic component of bone, can be synthesized on a large scale (Nayak, 2010) and has been widely applied in coatings on orthopedic implants (Oonishi, 1991), not only due to its excellent osteoinductivity and osteoconductivity but also given its ability to separate the implant surface from wear debris and regulate the immune response to accelerate tissue repair (Lahiri et al., 2011; Qin et al., 2018). Various methods, including sputter coating (Van Dijk et al., 1995), thermal spraying (Ong and Lucas, 1994), pulsed dynamic mixing (Yoshinari et al., 1994), laser ablation (Clèries et al., 2000), hot isostatic pressing (Wie et al., 1998), and dip coating (Shi et al., 2002), have been developed for HA coating. Nevertheless, fabrication process-related issues, such as harsh fabrication conditions and expensive instruments, make the doping of antibacterial molecules difficult. In addition, the intrinsic brittleness of HA normally leads to long-term instability of the coating, which impedes the osseointegration between implants and the bone (Lahiri et al., 2011).

Osseointegration is a complicated process, orchestrated by various biomolecular signals and cells in a spatiotemporally defined manner. Reactive oxygen species (ROS), considered as secondary regulators in a variety of biological processes, are among the key players in bone tissue regeneration and osseointegration, for which control of the ROS level is critical (Yao et al., 2019). After surgery and during the generation of wear debris, the environment around implants is under stress due to an excess of ROS, which is detrimental to osseointegration by activating osteoclasts and promoting macrophages to polarize to the M1 phenotype, a leading cause of implant failure (Kzhyshkowska et al., 2015). Eliminating the local stress associated with ROS by creating implants with an antioxidant profile was demonstrated to boost osteogenesis in both *in vitro* and *in vivo* models (Yang et al., 2019a).

**SCHEME 1:**
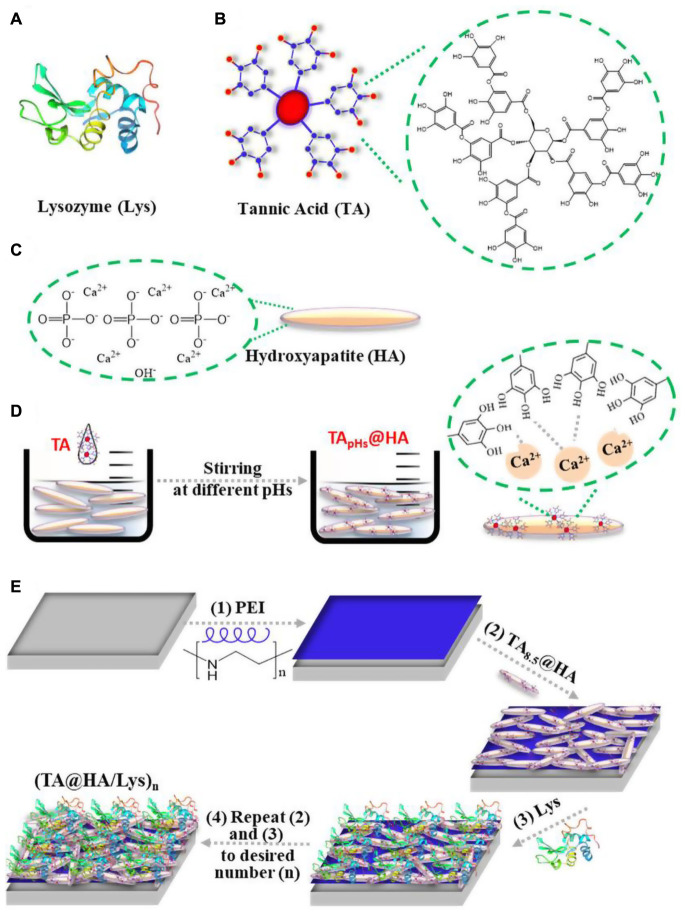
Schematic illustration of the molecular structure of **(A)** lysozyme (Lys), **(B)** tannic acid (TA), **(C)** hydroxyapatite (HA), **(D)** forming process of TA_*pHs*_@HA, and **(E)** (TA@HA/Lys)_n_ fabrication: (1) PEI was deposited on the substrate first, followed by the deposition of (2) TA@HA and (3) Lys, (4) repeating (2) and (3) with the desired number.

Lysozyme (Lys), as presented in Scheme 1A, with a molecular weight (Mw) of 14 kDa and an isoelectric point (IEP) of ∼11.2, is widespread in the secretions of some animal and human cells such as saliva, tears, and nasal mucus. In these secretions, it defends against microbial infection through cleaving peptidoglycan bonds, which are a major component of bacterial cell walls (Callewaert and Michiels, 2010). Owing to its specific mechanism of action against bacteria and high stability, Lys has been widely used for more than 80 years in pharmaceuticals to kill bacteria and in the food supply chain as a preservative (Juneja et al., 2012). Tannic acid (TA), as presented in Scheme 1B, one of the most common natural polyphenols, can be extracted from grape seed on a large scale. Owing to the multiple phenol groups in its molecular structure, TA exhibits strong antioxidant and ROS scavenging abilities by oxidizing phenol to quinone (Gülçin et al., 2010). To address these issues, here, utilizing the multimodal interactions of phenol groups with proteins (Akiyama et al., 2001) and their affinity to Ca^2+^ (Üçer et al., 2006), we demonstrated the integration of the beneficial profiles of TA, Lys, and HA into a coating applied on orthopedic implants *via* the layer-by-layer (LBL) technique. Compared to other coating techniques, the LBL technique was famous with its mild fabricating conditions, ample assemble materials, feasibility on different substrates with diverse shapes, etc. (Yang et al., 2019b). Most importantly, various bioactive molecules allowed to be incorporated into the LBL coatings for endowing the desired functionalities (Hammond, 2012). Our findings demonstrated that this composite film displayed strong antioxidant and antibacterial profiles, rapid attachment of cells [both mouse embryo osteoblast precursor cells (MC3T3-E1) and dental pulp stem cells (DPSCs)] in the early stage and their proliferation over a long period, enhanced osteogenesis of DPSCs and bone cells *in vitro*, and boosted bone regeneration and osseointegration *in vivo*.

## Materials and Methods

### Materials

Nano-sized HA (200 nm), TA, Lys, polyethylene imine (PEI, Mw ∼10 kDa), sulfuric acid (H_2_SO_4_), 30% hydrogen peroxide (H_2_O_2_), ascorbic acid (AA), sodium chloride (NaCl), tris(hydroxymethyl)aminomethane (Tris), disodium hydrogen phosphate (Na_2_HPO_4_), sodium dihydrogen phosphate (NaH_2_PO_4_), potassium dihydrogen phosphate (KH_2_PO_4_), and potassium chloride (KCl) were purchased from Shanghai Aladdin Biological Technology Co., Ltd., Shanghai, China, Rhodamine-FITC and 4’,6-diamidino-2-phenylindole (DAPI) were purchased from Sigma-Aldrich Life Sciences, Burlington, MA, United States. In addition, 4% paraformaldehyde, Triton X-100, β-glycerophosphate (β-GP), Alizarin Red S, and cetylpyridine chloride were purchased from Solarbio Life Sciences. Cell Counting Kit 8 (CCK-8) and Total Antioxidant Capacity Assay Kit [the Fluorescence recovery after photobleaching (FRAP) method] were purchased from Beyotime Biotechnology Institute, Shanghai, China. A silicon wafer and a round glass cover (13 mm in diameter) were washed with piranha solution (30% H_2_O_2_ and 70% H_2_SO_4_, v/v) at 90°C for 4 h, followed by a thorough cleaning with deionized water and storage in 75% alcohol before use.

### Preparation of HA Attached With TA

The mixture of TA (5 mg/ml) and AA (10 mM) containing a Bis-Tris or Tris buffer with defined pH of 4.0, 5.5, 7.0, and 8.5 was added into HA solution at a concentration of 50 mg/ml dispersed into the same buffers with the same pH. After incubating HA into TA solution with sonication for about 30 min, HA attached with TA (TA@HA) was collected by centrifugation at 6,000 rpm for 15 min. After water washing three times, the TA@HA was re-dispersed into the buffer with the pH set at 4.0, 5.5, 7.0, and 8.5 (defined as TA_pH_@HA) in the description below.

### Preparation of (TA@HA/Lys)_n_ Films

The (TA@HA/Lys)_n_ coatings were prepared on various substrates (cover glass, silicon wafer, and titanium rod) by the classic LBL technique. Briefly, PEI (1 mg/ml) was initially deposited onto the substrates for 20 min, after which the alternating deposition of TA@HA (for 10 min) and Lys (for 10 min) was carried out with the pH set at 4.0, 5.5, 7.0, and 8.5 until the desired cycle number (n) of layers was achieved [with samples hereafter defined as (TA@HA/Lys)_n__–__pH_]. During the LBL process, the substrates were thoroughly washed with water between each layer to remove free or loosely attached molecules.

### Cell Culture

Cells, including MC3T3-E1 cells and DPSCs, were cultured in an incubator at 37°C with 5% CO_2_. For the MC3T3-E1 cells, the culture medium Dulbecco’s Modified Eagle’s Medium (DMEM) contained 100 mg/ml streptomycin and 100 U/ml penicillin to avoid contamination. Then, 10% FBS was added to the medium as cell nutrition, which was refreshed every 2 days. The cells were passaged until the confluence reached 80–85%. For DPSCs, all conditions were the same as above, except that α-MEM was used instead of DMEM. Cells with fewer than five passages were used for all tests.

### Cell Morphology and Proliferation

Cover glasses coated with (TA@HA/Lys)_n_ were put into a 24-well plate and the uncoated slide was used as a control. After UV sterilization, 5 × 10^4^ cells (MC3T3-E1 cells and DPSCs) per well were seeded. For the cell proliferation assay, the seeding density was 2 × 10^4^ cells/well and CCK-8 was used to quantify the total viability of cells.

After incubation for the desired time, the cells were fixed with 4% paraformaldehyde. They were then treated with phosphate-buffered saline (PBS) supplemented with Triton X-100. The nuclei were stained with DAPI (blue), and the actin cytoskeleton was stained with TRITC Phalloidin (red) for morphological observation by fluorescence microscopy.

### Antioxidant Assay

The Total Antioxidant Capacity Assay Kit employing the FRAP method was used to test the antioxidant capacity of (TA@HA/Lys)_n_, in accordance with the manufacturer’s instructions. To measure the cellular antioxidant capacity of (TA@HA/Lys)_n_, 4 × 10^4^ cells/well were seeded. After 24 h of culture, 10 μM H_2_O_2_ as a peroxide stimulator was added. After another set time, the cells were fixed, stained, and imaged as mentioned in the section “Cell morphology and proliferation.”

### Osteoblast Mineralization and Alkaline Phosphatase Activity

A total of 4 × 10^4^ MC3T3-E1 cells per well were seeded on (TA@HA/Lys)_n_ and cultured in a normal medium with/without osteogenic inducers (β-GP and AA). After 14 days, Alizarin Red S was used to detect calcium (Ca) deposition. The absorbance of Alizarin Red S at 570 nm was determined by using a microplate reader. Briefly, the samples were fixed with 4% paraformaldehyde and treated with 0.1% Alizarin Red S solution (pH 4) for staining. After thoroughly washing the free Alizarin Red S using PBS and water, 300 μl of 10% cetylpyridine chloride solution was added to elute Alizarin Red S attached to Ca nodules. A total of 100 μl of dye solution was collected, and the absorbance at 590 nm was determined by using a microplate reader to quantify the Ca nodules.

Alkaline phosphatase (ALP) activity was measured at 7 and 14 days after cell seeding, using an ALP assay kit, in accordance with the manufacturer’s instructions. The ALP content was determined based on the absorbance at 405 nm.

### Real-Time Quantitative PCR

Real-time quantitative PCR (qRT-PCR) was used to determine the expression levels of the osteogenic-related genes, runt-related transcription factor 2 (Runx2), osteonectin (ON), and osteocalcin (OCN). DPSCs were seeded at 2 × 10^4^ cells/well on (TA@HA/Lys)_n_. After 7 and 14 days of culture, total RNA was extracted and reverse transcribed by using the PrimeScript RT reagent kit to produce the first strand of complementary DNA (cDNA). β-actin was selected as a gene for normalization, and the data were analyzed by using the 2 ^–ΔΔ*Ct*^ method. The results were normalized by using the mean value of the control group.

### *In vivo* Test

The *in vivo* test was performed on New Zealand rabbits with a mean weight of 2.3 kg and ages ranging from 5 to 6 months. About 12 rabbits were randomly divided into six groups, (TA@HA/Lys)_n_ (*n* = 2, 4) for 4 and 8 weeks of implantation, along with the (TA@HA/Lys)_0_ group that received no coating and was used as a control.

A round bone defect with a diameter of 5 mm and a depth of 5 mm was artificially created at the femoral condyle, into which a titanium rod with a (TA@HA/Gel)_n_ coating was implanted. After the 4th and 8th week, the rabbits were sacrificed and the corresponding femur was removed and immersed in 4% paraformaldehyde at 4°C for more than 1 month.

Micro-CT was used to image the distal femur with the following parameters: voltage 80 kV, current 300 mA, and 360° rotation with rotation steps of 0.5°. Focus was placed on the new bone formation between the bone tissue and implants. Briefly, the selected region of interest (ROI) around the distal femur was reconstructed by the CT-Volume (CTVol) software, by which the bone volume (BV) and total volume (TV) of bone were determined.

### Antibacterial Test

Bacteria (*Staphylococcus aureus* and *Escherichia coli*) were collected by centrifugation and resuspended in PBS buffer solution at 1 × 10^7^ bacteria/ml. The (TA@HA/Lys)_n_ samples and the control (no coating) were placed in a 24-well plate, to which 1 ml of the above bacterial solution was added. After incubation for 6 h at 37°C, all samples were washed with PBS three times, dried under vacuum, and imaged by field-emission scanning electron microscopy (FSEM).

### Characterization and Data Analysis

Instruments, including a FESEM (SU8010; Hitachi, Tokyo, Japan), an atomic force microscope (AFM) (Dimension ICON; Bruker, Santa Barbara, CA, United States), an x-ray photoelectron spectrometer (XPS) (ESCALAB 250; Thermo Fisher Scientific, Waltham, MA, United States), and an ellipsometer (EX2; ELLITOP, Beijing, China), were used for material-related characterization.

A confocal laser scanning microscope (CLSM) (A1; Nikon, Tokyo, Japan) and a fluorescence microscope (DMi8; Leica, Heidelberg, Germany) with a live cell workstation were used for cell imaging. A microplate reader (Varioskan LUX; Thermo Fisher Scientific, Waltham, MA, United States), a quantitative real-time fluorescence quantitative PCR (qRT-PCR) (LightCycler^®^96; Roche Applied Science, Mannheim, Germany), and a high-resolution micro-CT (Skyscan1176; Bruker, Billerica, MA, United States) were used for biological characterization.

All data are reported as the average of at least three duplicates, with the error bar indicating the SD. Statistical analysis was performed by the *t*-test, and significance was noted as “^∗^,”“^∗∗^,”“^∗∗∗^,”and “^****^” for *p* < 0.05, *p* < 0.01, *p* < 0.001, and *p* < 0.0001, respectively.

## Results and Discussion

### Adsorption of TA Onto HA (Abbreviated as TA@HA)

**FIGURE 1:**
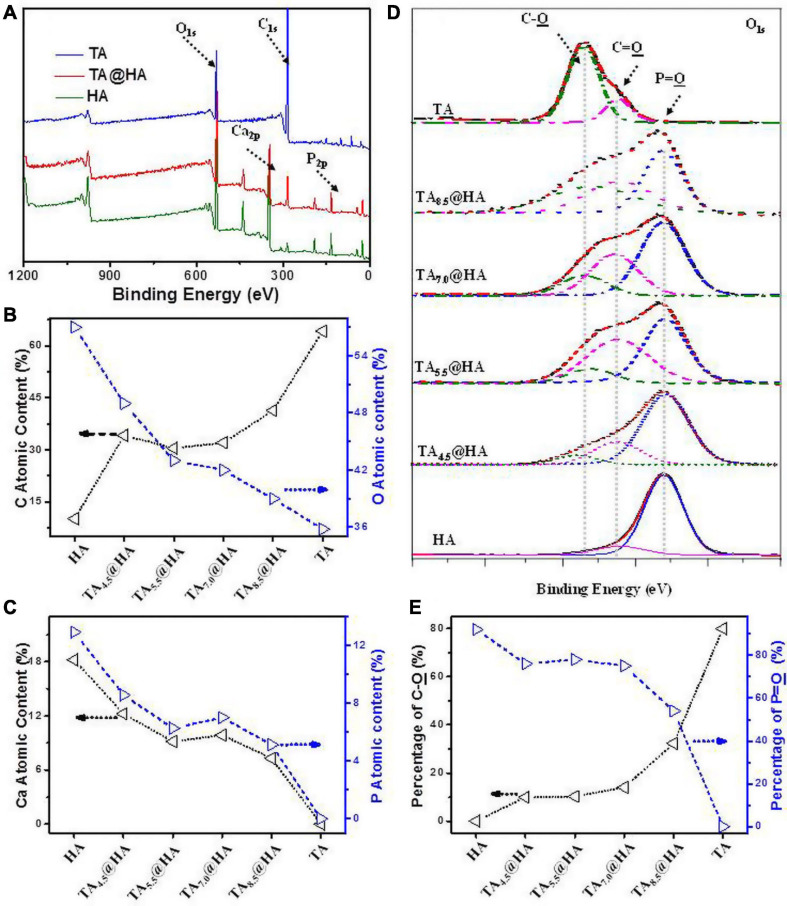
**(A)** X-ray photoelectron spectrometer (XPS) spectra of tannic acid (TA), TA_–__8.5_@HA, and hydroxyapatite (HA). **(B)** Carbon **(C)** and oxygen (O) contents and (C) Calcium (Ca) and phosphorus (P) contents in TA, TA_–__pH_@HA, and HA. **(D)** Peak fitting in O_1s_ of XPS spectra of TA, TA_–__pH_@HA, and HA. **(E)** C-O and P = O contents in TA, TA_–__pH_@HA, and HA relative to the total O calculated from **(D)**. The line in all figures was used for eye guidance.

After incubating HA (Scheme 1C) in TA solutions, the adsorption of TA onto HA (TA@HA) was performed at different pHs (Scheme 1D). Considering that the thickness of the adsorbed TA was on the nanoscale, XPS, a surface-sensitive technique, was employed to detect the attachment. Typical XPS spectra of HA, TA, and TA@HA are displayed in Figure 1A, where no significant difference was observed between HA and TA@HA at a first glance because all peaks of O_1s_ (532 V), C_1s_ (285 eV), Ca_2p_ (347 eV), and P_2p_ (133 eV) were detected. The appearance of C_1s_ in HA was attributed to the doping of carbonate into HA during its synthesis. However, upon a detailed analysis, the differences between HA and TA@HA could be traced in the elemental compositions of carbon (C), oxygen (O), Ca, and phosphorus (P) (Wei et al., 2021). As shown in Figure 1B, the C atomic content greatly increased from the initial stage as 10.1% in HA to 41.3% in TA_8.5_@HA, whereas the O atomic content decreased from 57 to 39%. Although the C and O atomic contents were slightly far from those of TA (64.1% C and 35.8% O), the tendencies of content change closing to TA provided strong evidence of successful attachment of TA. The decreased atomic contents of elements (Ca and P) belonging to HA (Figure 1C) further supported the deposition of TA onto HA. In addition, the ratio of Ca to P was around 1.58 for all tested TA_pH_@HA samples, a value very close to 1.60 of the original HA, confirming the reliability of the XPS data. From Figures 1B,C, the attachment of TA was clearly pH-dependent, with more TA occurring at higher pH. To obtain more clues about the pH-dependence of TA@HA forming, a detailed analysis of O_1s_ was carried out. Owing to the different chemical environments, the peak of O_1s_ centered at 532 eV was split into several peaks, as displayed in Figure 1D, such as P = O (531.2 eV), C = O (532.5 eV), and C-O (533.3 eV). As shown in Figure 1D, the P = O peak could be only found in HA, whereas the C-O peak was only in TA. The P = O and C-O peaks were considered to originate from HA and TA, respectively, and their levels could represent the quantities of HA and TA. As shown in Figure 1E, the percentages of C-O and P = O relative to total O, along with the continuous decrease of P = O and the increase of C-O with increasing pH, strongly confirmed the greater deposition of TA, which was consistent with the results of the above elemental analysis. The pH-dependent deposition of TA was attributed to pH-dependent coordinative interaction with the metal surface and pH-dependent self-polymerization of TA (Ringwald and Ball, 2015). Considering that pH 8.5 was associated with the highest level of adsorbed TA, this condition was selected to prepare TA@HA in the subsequent experiments.

### Fabrication of TA@HA/Lys Coatings

**FIGURE 2:**
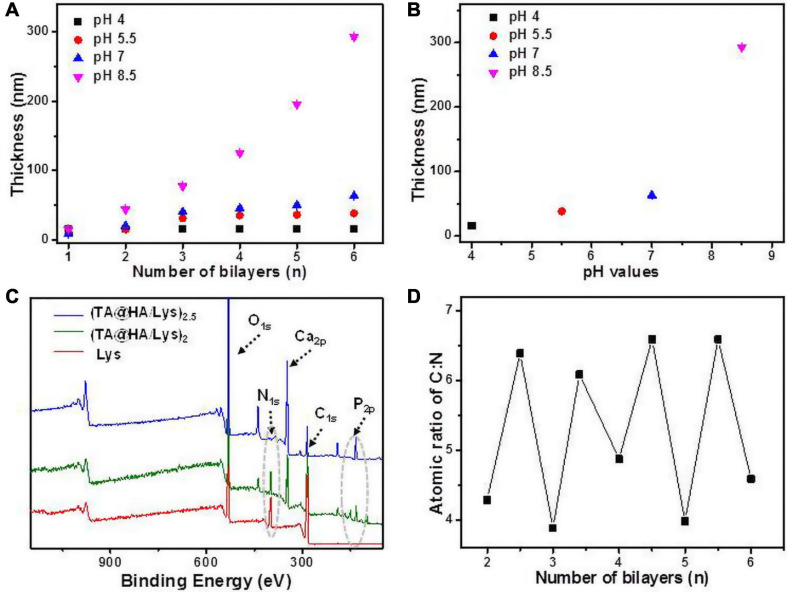
**(A)** The thickness of (TA@HA/Lys)_n__–__pH_ deposited at various pH values plotted as the function of a number of bilayers. **(B)** The thickness of (TA@HA/Lys)_6__–__pH_ constructed at different pHs. **(C)** XPS spectra of lysozyme (Lys), (TA@HA/Lys)_2–8__.5_, and (TA@HA/Lys)_2.__5–8__.5_. **(D)** Elemental content ratio of C:N in (TA@HA/Lys)_n__–__8.5_ as the function of a number of bilayers. The line in all figures was used for eye guidance.

From (TA@HA/Lys)_n__–__pH_ preparation, as shown in Scheme 1E, beginning with the deposition of PEI followed by the recycled deposition of TA@HA and Lys, TA@HA/Lys coating was obtained after the desired number of repeat cycles. The thickness of (TA@HA/Lys)_n__–__pH_ was monitored with the increasing number of deposition cycles. To optimize the LBL conditions and considering the pH-dependent attachment of TA onto HA, the growth of (TA@HA/Lys)_n_ at different pH levels was evaluated. As displayed in Figure 2A, (TA@HA/Lys)_n__–__pH_ exhibited continuous growth but strong pH dependence, where higher pH led to a thicker coating. The thickness of (TA@HA/Lys)_6__–__pH_ (Figure 2B) was about 290 nm, as produced at pH 8.5, which then decreased in the following order: 68 nm at pH 7, 38 nm at pH 5.5, and 15 nm at pH 4. As control experiments, LBL for TA and Lys was performed under the same condition. The thickness of (TA/Lys)_6__–__pH_ (Supplementary Figure 1) was only 58 nm at pH 8.5, 51 nm at pH 7, 25 nm at pH 5.5, and 18 nm at pH 4, which were comparable to those of (TA@HA/Lys)_6__–__pH_ at other pH values except pH 8.5. Clearly, such a substantial thickness at pH 8.5 with a few cycles suggested that the main contributor to thickness was HA (average size of around 200 nm), not the complexation of TA and Lys. The slow increase of (TA@HA/Lys)_6–4__,5.5,*and*7_ was possibly attributable to free TA in the TA@HA solution during LBL, which was detached from TA@HA. The above phenomenon should be attributed to solution pH switching from 8.5 to 4, 5.5, and 7. Coordinative interaction and self-polymerization of TA were strongly dependent on the pH, so TA could free itself again from TA@HA when the pH switched from the deposition at 8.5 to LBL at 4, 5.5, and 7. The detached emulsion-like TA molecules occupied the sites in Lys, which were previously bonded to TA@HA. As reported previously (Yang et al., 2019b), there were multiple modes of interaction between Lys and TA, which actuated the coating growth. It can thus be readily imagined that TA acted as a glue to stick HA and Lys together. Since the thickest coating was obtained at pH 8.5, these conditions were used for preparing (TA@HA/Lys)_n_ coatings.

The success of coating generation was further confirmed by XPS. As shown in Figure 2C, compared with the findings for TA@HA, the appearance of nitrogen signals and the maintenance of Ca and P signals suggested the presence of Lys and HA in (TA@HA/Lys)_2*and*2.5_. However, the signal of nitrogen in (TA@HA/Lys)2.5 was much weaker than that in (TA@HA/Lys)_2_. In Figure 2D, the signal ratio of C to N is plotted against the number of bilayers. The fluctuation of the plot strongly supports the LBL deposition.

### Morphology of (TA@HA/Lys)_n_ Coatings

**FIGURE 3:**
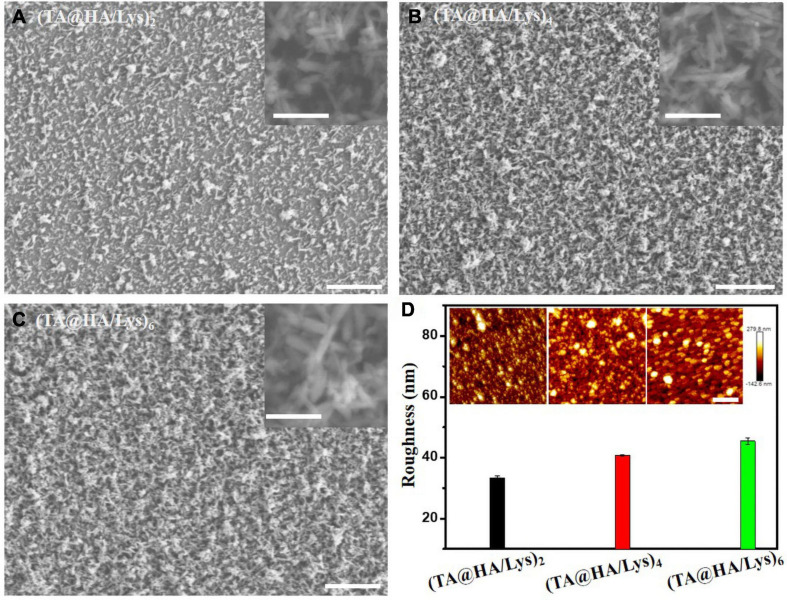
SEM images of **(A)** (TA@HA/Lys)_2_, **(B)** (TA@HA/Lys)_4_, and **(C)** (TA@HA/Lys)_6_, and the corresponding magnified images in the insets of the upper right. **(D)** Surface roughness calculated from the atomic force microscope (AFM) images of (TA@HA/Lys)_2_ and (TA@HA/Lys)_4_ coatings, the insets are the corresponding AFM images. The scale bars in **(A–C)** are 20 μm (200 nm in the insets). The scale bar in **(D)** is 2 μm.

The surface morphology of materials has a great influence on cell viability and cell behavior (Wang et al., 2019). To observe the film surface morphology, SEM and AFM were used to characterize it. Under SEM, the HA particles (Figures 3A–C) were acicular, with a size of a few hundred nanometers and an irregular distribution. As the number of layers increasing, the density of HA/Lys films increased. The substrate was gradually covered along with the increase of thickness, which proved the successful deposition of (TA@HA/Lys)_n_. All (TA@HA/Lys)_n_ coatings with different cycle numbers were evenly applied, with a distributed porous structure, which was constructed by the random deposition of TA@HA (Figures 3A–C). Under AFM, the measured root mean square (RMS) roughness of (TA@HA/Lys)_2_, (TA@HA/Lys)_4_, and (TA@HA/Lys)_6_ films were 32.3 ± 0.7, 38.75 ± 0.25, and 45.4 ± 1.12, respectively (Figure 3D). With the increase in the number of film layers, the RMS roughness increased continuously, which was due to the irregular accumulation of HA particles and the existence of pores in the film plane. The increased roughness might contribute to the nonlinear growth of this composite coating (Jiang et al., 2014). In addition, the coating was stable in the cell culture medium, as evidenced by no obvious changes of thickness and morphologies after incubating into the cell culture medium for more than 3 weeks.

### Interaction Between Cells and (TA@HA/Lys)_n_ Coatings

Interaction between cells and biomaterial is a central issue in tissue engineering research (Przekora, 2019), which is based on initial adhesion between cells and a material surface. To achieve successful implantation, the cells must first appropriately adhere to the surface of a biomaterial, laying the foundation for subsequent differentiation, migration, and proliferation (Bacakova et al., 2011). Besides, long-term cell proliferation is critical to tissue generation and integration with surrounding tissues (Zhao et al., 2013). The attachment and spread of cells onto implants as early as possible is highly beneficial to the integration of tissues and cells and reduce the possibility of bacterial adhesion (Godoy-Gallardo et al., 2016). Two cell lines, the osteoblastic cell line MC3T3-E1 and DPSCs, were chosen to investigate the attachment and spread on (TA@HA/Lys)_n_ coatings at an early stage, as well as long-term proliferation. MC3T3-E1 cells have the capacity to differentiate into osteocytes and osteoblasts (Choi et al., 1996), which have been demonstrated to be a classic *in vitro* cellular phenotype to form calcified bone tissue. DPSCs have immunophenotypes similar to those of bone marrow mesenchymal stem cells (Gronthos et al., 2002), and form mineralized nodules, with abundant sources, no rejection reactions, and no ethical issues.

**FIGURE 4:**
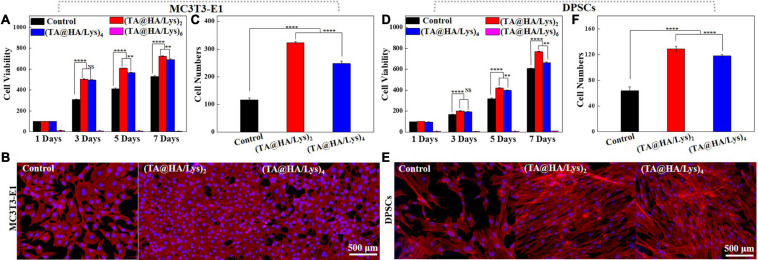
**(A)** Cell viability of mouse embryo osteoblast precursor cells (MC3T3-E1) cultured onto control, (TA@HA/Lys)_2_, (TA@HA/Lys)_4_, and (TA@HA/Lys)_6_ at 1, 3, 5, and 7 days. **(B)** Fluorescence images and **(C)** cell numbers of MC3T3-E1 cells cultured onto control, (TA@HA/Lys)_2_, (TA@HA/Lys)_4_, and (TA@HA/Lys)_6_ at day 5. **(D)** Cell viability of dental pulp stem cells (DPSCs) cultured onto control, (TA@HA/Lys)_2_, (TA@HA/Lys)_4_, and (TA@HA/Lys)_6_ at 1, 3, 5, and 7 days. **(E)** Fluorescence images and **(F)** cell numbers of DPSCs cultured onto control, (TA@HA/Lys)_2_ and (TA@HA/Lys)_4_ at day 5. All images were using the same scale bar. **p < 0.01, ****p < 0.0001.

To investigate the proliferation of MC3T3-E1 cells and DPSCs on (TA@HA/Lys)_n_ coatings, these cells were cultured on glass slides with the abovementioned coatings marked as (TA@HA/Lys)_2,4_,_*and*6_ and without coating as the control group. Cellular viability and proliferation profiles were determined by using (CCK-8) on days 1, 3, 5, and 7. The viability of MC3T3-E1 cells is shown in Figure 4A, indicating that the cellular viability of all samples increased with increasing incubation time, and that the cellular viability on (TA@HA/Lys)_2*and*4_ was higher than that of the control group throughout the testing period, but was very low on (TA@HA/Lys)_6_. This suggested that there were more cells on (TA@HA/Lys)_2*and*4_ than in the control group, and no cells could survive on (TA@HA/Lys)_6_. In addition, the (TA@HA/Lys)_2_ samples exhibited the highest cell proliferation among all tested samples. Owing to the limited ability of (TA@HA/Lys)_6_ to induce cell proliferation, only (TA@HA/Lys)_2*and*4_ coatings were used in subsequent research.

On day 5, the cells were stained and imaged. Figure 4B clearly shows that the numbers of MC3T3-E1 cells on (TA@HA/Lys)_2*and*4_ coatings were greater than in the control, with the highest number being found on (TA@HA/Lys)_2_, as further supported by statistics on cell numbers (Figure 4C). For DPSCs, very similar results (cell viability in Figure 4D, fluorescent images of cells on day 5 in Figure 4E, and statistical results of cell numbers in Figure 4F) to those of MC3T3-E1 were found. The cellular proliferation rate on (TA@HA/Lys)_n_ was clearly significantly higher than that of the control group.

**FIGURE 5:**
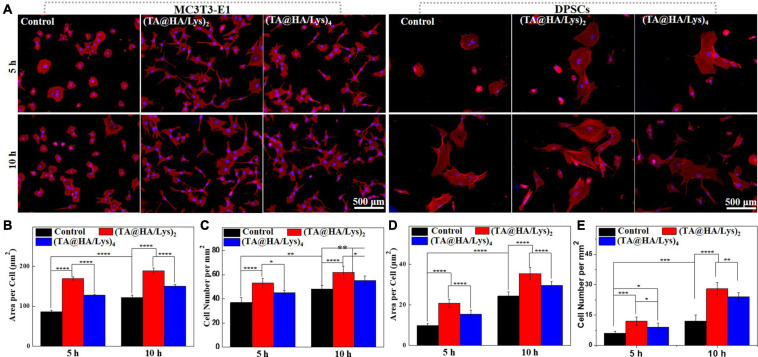
**(A)** Morphologies of MC3T3-E1 cells and DPSCs cultured on glass coverslips as control (left row), (TA@HA/Lys)_2_ (middle row), and (TA@HA/Lys)_4_ (right row) substrates for 5 and 10 h. **(B)** Statistical cell area per cell and **(C)** cell number per mm^2^ of MC3T3-E1 cells. **(D)** Statistical cell area per cell and **(E)** cell number per mm^2^ of DPSCs. All images were using the same scale bar. *p < 0.05, **p < 0.01, ***p < 0.001, ****p < 0.0001.

To investigate the attachment and spread of cells at an early stage, MC3T3-E1 cells and DPSCs were inoculated into (TA@HA/Lys)_n_ coatings, and fluorescence staining images (Figure 5A) were taken at 5 and 10 h. The membrane-free round cover glass was used as a control (Figure 5A). It was apparent that MC3T3-E1 cells and DPSCs were attached to (TA@HA/Lys)_n_ films and exhibited spreading. At 5 h (Figure 5A, top), cells on (TA@HA/Lys)_n_ were already in the early stage of spreading, with pseudopodia appearing and cells adopting a spindle type, while most cells in the control group were round. At 10 h (Figure 5A, bottom), cells on (TA@HA/Lys)_n_ coatings in the control group had all spread, but (TA@HA/Lys)_2_ had the most cells attached to the surface and appeared to have the largest cellular area (Figure 5A, left). Further analysis by the Image J software showed that, compared with the levels in the control group, more cells attached to (TA@HA/Lys)_n_ coatings and there was a greater cellular area per cell (Figures 5B,C), suggesting that the MC3T3-E1 cells had spread well on (TA@HA/Lys)_n_. From Figures 5B,C, (TA@HA/Lys)_2_ displayed the best performance regarding cell spread. For DPSCs attached to (TA@HA/Lys)_*n*,_ a clear cell morphology was also exhibited (Figure 5A, right). At 5 h (Figure 5A, top), most of the DPSCs in the control group were round, and a small number of cells appeared to be at the initial stage of spread. A few DPSCs were round on (TA@HA/Lys)_n_ coatings, and most of the cells were in the early stage of spread. At 10 h (Figure 5A, bottom), the cells on (TA@HA/Lys)_n_ coatings and the control group started spreading, and the number of attached cells on (TA@HA/Lys)_n_ coatings increased. Compared with the levels in the control group, more cells were attached to (TA@HA/Lys)_n_ coatings and there was a greater cellular area per cell (Figures 5D,E), suggesting that DPSCs preferred (TA@HA/Lys)_n_ coatings. Again, (TA@HA/Lys)_2_ displayed the best cell spread performance from evaluating both cell number and cellular area per cell. Taking these findings together, (TA@HA/Lys)_n_ coatings have the ability to promote not only the adhesion and spread of cells in the early stage, but also their proliferation in the long term. The slight differences shown in these results could be based on the differences in cellular phenotype and roughness of the coating surface.

### Antioxidant Activity and Sterilization Ability

Around implants, there is significant stress due to the abundance of ROS (Atashi et al., 2015), caused by a variety of factors, such as blood clot after injury or surgery (Barrett et al., 2018), and inflammation induced by debris from wear that has peeled away from implants over a long period (Lei et al., 2015). In addition, this stressed ROS-rich environment cannot be easily remedied by reductive biomolecules produced by the cell to balance ROS levels (Yao et al., 2019). Therefore, endowing implants with antioxidant properties would be greatly beneficial to their success (Shuai et al., 2018). Antibacterial properties are also crucial to implants (Stewart and William Costerton, 2001), since infections frequently occur around them, being a leading cause of implant failure. A large number of pyrocatechol and catechol groups, which confer antioxidant and antibacterial properties, are distributed in the molecular surface of TA. Besides, incorporating Lys well-known for its antibacterial activity through an LBL technique could make the coating a promising feature for sterilization.

**FIGURE 6:**
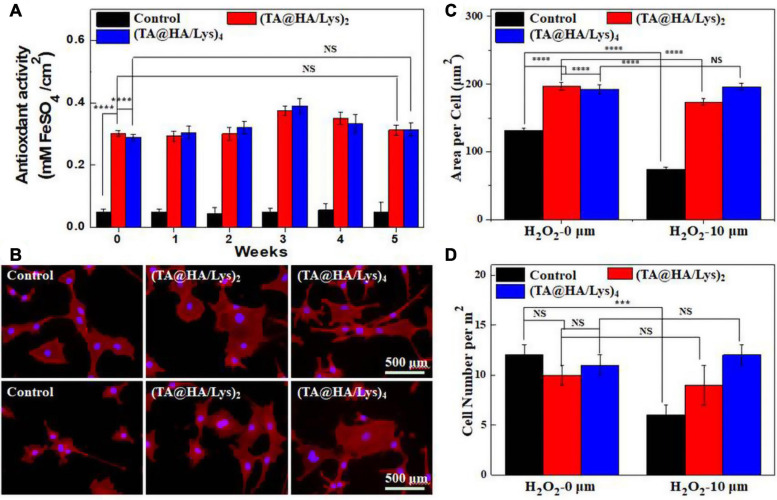
**(A)** Antioxidant activities of glass coverslips as control, (TA@HA/Lys)_2_, and (TA@HA/Lys)_4_ films evaluated by the fluorescence recovery after photobleaching (FRAP) method. **(B)** Fluorescence microscopy images of MC3T3-E1 cells cultured on glass coverslips as control, (TA@HA/Lys)_2_, and (TA@HA/Lys)_4_ substrates before (top) and after (bottom) 10 μM hydrogen peroxide (H_2_O_2_) stimulation. **(C)** Cell area per cell and **(D)** cell number per mm^2^ of MC3T3-E1 cells on the above substrates were determined without/with 10 μM H_2_O_2_ stimulation. All images were using the same scale bar. ***p < 0.001, ****p < 0.0001.

The antioxidant properties of (TA@HA/Lys)_n_ coatings were evaluated at different time intervals (0, 1, 2, 4, 8, and 10 weeks) by the FRAP method, with the standard curve displayed (Supplementary Figure 2). The test results (Figure 6A) showed that the antioxidant activities of all (TA@HA/Lys)_n_ coatings were much higher than that of the control group, showing no obvious decrease within the tested time period. There was good maintenance of antioxidant activity of coatings with the passing of time, which was of great significance for the subsequent *in vivo* experiments and future clinical applications (Asghar et al., 2021). The antioxidant properties of (TA@HA/Lys)_n_ coatings in the cellular level were investigated by supplying H_2_O_2,_ a well-known ROS producer that is widely used to generate excessive ROS in models of ROS-related stress (Rigoulet et al., 2011). Before and after the stimulation with H_2_O_2_, MC3T3-E1 cells were stained and imaged. As shown in Figure 6B, the cells spread out well without H_2_O_2_ stimulation (top panel) while they visibly shrank after the treatment with 10 μM H_2_O_2_ (bottom panel) in the control group, and displayed no significant morphological changes after the treatment with 10 μM H_2_O_2_ on (TA@HA/Lys)_n_ coatings. Area per cell and cell number per mm^2^ were statistically analyzed, with the results displayed in Figures 6C,D, respectively. In the control group, both cell area and cell number greatly decreased after the stimulation with 10 μM H_2_O_2_ while they showed no significant differences in (TA@HA/Lys)_n_ coatings after 10 μM H_2_O_2_ stimulation, independent of the number of coatings. The above findings indicated that (TA@HA/Lys)_n_ coatings possess the strong antioxidant ability and were conducive to the proliferation of cells.

**FIGURE 7:**
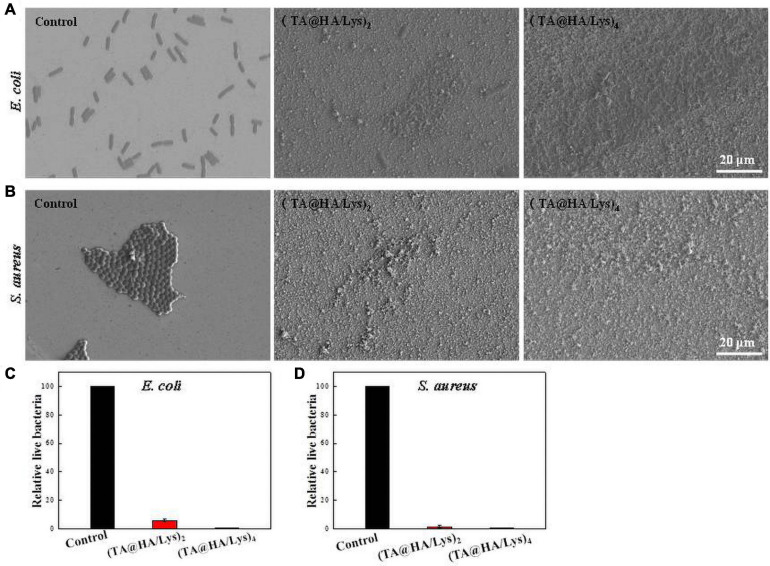
SEM images of **(A)** *Escherichia coli* and **(B)** *Staphylococcus aureus* after exposure to control (glass coverslips, left row), (TA@HA/Lys)_2_ (middle row), and (TA@HA/Lys)_4_ (right row) coatings. Relative live bacteria number of **(C)** *E. coli* and **(D)** *S. aureus* in above films (the control group as a reference). All images were using the same scale bar.

Sterilization ability was evaluated by seeding two representative bacteria (Gram-negative bacteria, *E. coli*, and Gram-positive bacteria, *S. aureus*) onto (TA@HA/Lys)_n_ coatings. Owing to the porous structure of (TA@HA/Lys)_n_ coatings, the dye-staining live/dead bacteria tended to be trapped in the surface, which made it very difficult to evaluate sterilization efficiency. However, the sterilization efficiency of (TA@HA/Lys)_n_ coatings could be confirmed by SEM images. As displayed in Figure 7A (*E. coli*) and Figure 7B (*S. aureus*), in comparison with the control group, very few bacteria with an intact membrane remained and bacterial corpses were exposed on (TA@HA/Lys)_n_ coatings everywhere, through lysis, breakage, deformation, and collapse of the bacterial membrane. *Via* statistical analysis, the number of live bacteria relative to that in the control group was determined and plotted, as shown in Figures 7C,D. The findings suggested that (TA@HA/Lys)_n_ coatings had strong sterilization ability against both *E. coli* and *S. aureus*. Compared with killing *E. coli*, (TA@HA/Lys)_n_ coatings appeared to exhibit a more formidable ability to kill *S. aureus*. In addition, (TA@HA/Lys)_n_ coatings with more layers exhibited strong sterilization ability due to more antibacterial components incorporated into the coatings. Such high efficiency of sterilization exhibited by (TA@HA/Lys)_n_ should be attributable to the multiple antibacterial pathways of TA and Lys. Lys kills bacteria through catalyzing the hydrolysis of 1,4-β linkages between *N*-acetyl-D-glucosamine and *N*-acetylmuramic acid in the bacterial wall (Masschalck and Michiels, 2003), while TA molecules achieve sterilization by binding proteins inside or on the membrane of bacteria (Akiyama et al., 2001). In addition, the needle-like structure of HA and the rough porous surface of (TA@HA/Lys)_n_ coatings should be other factors contributing to such strong sterilization ability.

### Osteoblast Mineralization and Activity of ALP

**FIGURE 8:**
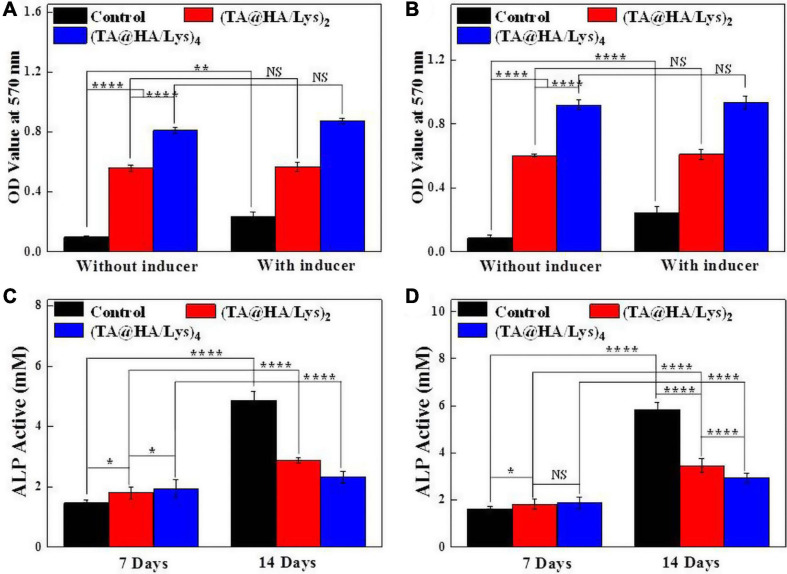
OD values at 570 nm after extracting the Alizarin Red S stained for evaluating the osteoblast mineralization of **(A)** MC3T3-E1 cells and **(B)** DPSCs cultured on glass coverslips as control, (TA@HA/Lys)_2_ and (TA@HA/Lys)_4_ films with/without induction solution for 14 days. Alkaline phosphatase (ALP) activities of **(C)** MC3T3-E1 cells and **(D)** DPSCs cultured on glass coverslips as control, (TA@HA/Lys)_2_ and (TA@HA/Lys)_4_ films for 7 and 14 days. *p < 0.05, **p < 0.01, ****p < 0.0001.

In the human body, HA is mainly distributed in the bones and teeth. During the calcification process of bone cells, HA can provide a crystal nucleus for bone calcification and the osteogenesis of bone cells, and plays a role in bone conduction (Oonishi, 1991). (TA@HA/Lys)_n_ coatings had a good effect on cell proliferation, so the assembled films were co-cultured with bone cells for 14 days to detect the mineralization of osteoblasts. Ca deposition is considered as an indicator of mineralization, and the level of cell mineralization can be represented by the results of Alizarin Red S staining. The MC3T3-E1 cells were cultured in an osteogenic medium with or without the osteogenic induction for 14 days. The characteristic absorbance of Ca at 570 nm was measured to evaluate the degree of mineralization. In the control group (Figure 8A, black histograms), there was a significant difference in OD (optical density) values at 570 nm between the samples with and without the bone inducer, suggesting that the Ca deposition increased significantly in the presence of the bone inducer. However, for (TA@HA/Lys)_n_ coatings (Figure 8A, red and blue histograms), there was no significant difference in OD values between the cases when the inducer was added or not; meanwhile, the Ca deposition was much higher than that in the corresponding control group. Although the Ca deposition in the (TA@HA/Lys)_2_ coating film was lower than that in the (TA@HA/Lys)_4_ film, the deposition of (TA@HA/Lys)_4_ and (TA@HA/Lys)_2_ were 8.3 and 5.8 times higher than that in the control group without an inducer, respectively. In comparison with the control films to which an inducer had been added, differences in the fold in the Ca deposition were reduced to 2.4 and 3.7 for (TA@HA/Lys)_2_ and (TA@HA/Lys)_4_ coatings, respectively, which should be attributable to the increased Ca deposition in the control group. The same experiments were carried out on DPSCs, for which the data are presented in Figure 8B. Some very similar phenomena regarding the Ca deposition were found, which supported the above results on the Ca deposition of MC3T3-E1 cells. Meanwhile, the difference between (TA@HA/Lys)_n_ coatings and the control became much greater than that found for MC3T3-E1 cells. Especially for the case without an inducer, the Ca deposition onto (TA@HA/Lys)_4_ coating was 10.8-fold that in the control, while it was 7.1-fold that for the (TA@HA/Lys)_2_ coating. With an inducer, the fold increases [2.3 and 3.8 for (TA@HA/Lys)_2_ and (TA@HA/Lys)_4_ coatings, respectively] were very close to those in MC3T3-E1 cells. These results indicated that (TA@HA/Lys)_n_ coatings have the ability to promote cellular mineralization, which is enhanced with the increase of deposited materials. Most importantly, the Ca deposition ability exhibited by (TA@HA/Lys)_n_ coatings could be critical to *in vivo* bone-related tissue repair (Gorgieva et al., 2017), especially for some sites or some patients in which repair is not possible due to a lack or very low concentration of osteogenic inducer.

Alkaline phosphatase is the most widely recognized early marker of osteoblast differentiation (Beck et al., 1998), as a typical protein product of the osteoblast phenotype and osteoblast differentiation. (TA@HA/Lys)_n_ coatings have an osteogenic mineralization effect on MC3T3-E1 cells, and DPSCs have the ability to differentiate into osteoblasts as well. Hence, the effects of (TA@HA/Lys)_n_ coatings on the osteogenic differentiation of MC3T3-E1 cells and DPSCs were evaluated, and ALP expression was quantitatively analyzed after osteogenic induction for 7 and 14 days on the cultured cells (Figures 8C,D). The ALP results showed that, on the 7th day, the expression of ALP in the control group was lower than that in (TA@HA/Lys)_n_ coatings, and the expression in (TA@HA/Lys)_2_ was lower than that in (TA@HA/Lys)_4_. On day 14, the ALP activity in the control group peaked. The expression of ALP in DPSCs exhibited a similar trend as that in MC3T3-E1 cells, except for some specific values. The ALP activity of HA/Lys films significantly decreased, demonstrating that DPSCs differentiated into mature osteoblasts, and HA/Lys films possessed the ability to promote cell differentiation. The ability to promote cell differentiation may be positively correlated with HA concentration.

### Expression Levels of Genes Related to Osteogenic Differentiation

**FIGURE 9:**
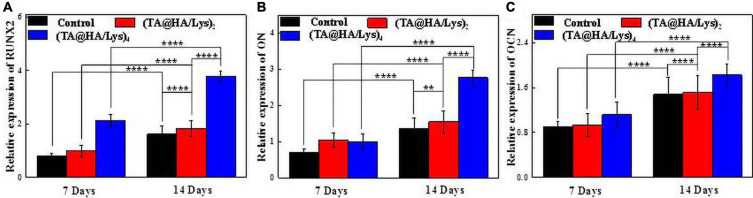
Expressions of genes in relation to osteogenic differentiation for DPSCs being inductively cultured on the (TA@HA/Lys)_2_ and (TA@HA/Lys)_4_ substrates and glass coverslips as control: **(A)** runt-related transcription factor 2 (Runx2), **(B)** osteonectin (ON), and **(C)** osteocalcin (OCN). **p < 0.01, ****p < 0.0001.

The gene Runx2 encodes a transcription factor essential for osteoblast differentiation (Komori, 2006). OCN is a bone tissue-specific protein (Frendo et al., 1998), which is mainly expressed in osteoblast differentiation. Moreover, ON, a glycoprotein attached to collagen (Termine et al., 1981), is abundant in the bone matrix and plays an important role in initiating the mineralization and promoting the deposition of minerals on collagen components. These three representative genetic markers associated with the osteoblastic differentiation of DPSCs at different stages were analyzed by qRT-PCR after osteogenic differentiation on (TA@HA/Lys)_n_ coatings for 7 and 14 days (Figures 9A–C). Compared with that at 7 days, gene expression levels on the 14th day in both the control group and (TA@HA/Lys)_n_ coatings increased. In comparison with the findings in the control group, (TA@HA/Lys)_n_ coatings significantly promoted gene expression, and the expression levels of these three genes in (TA@HA/Lys)_4_ film were the highest. This indicated that (TA@HA/Lys)_n_ coatings have the ability to promote osteogenic gene expression and their regulatory effect on osteogenic differentiation is to significantly increase it. This may be due to the ability of HA to promote cell differentiation, which increases with increasing concentration.

### *In vivo* Test

**FIGURE 10:**
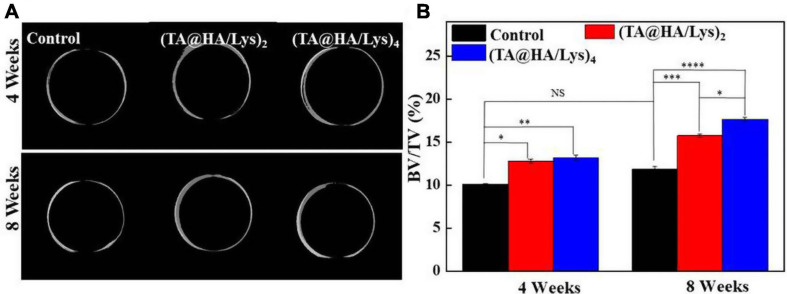
**(A)** Bone regeneration images in the rabbit femur were reconstructed by the CT-Volume (CTVol) software in three-dimensional (3D) model, when titanium rods with a blank layer as control, (TA@HA/Lys)_2_ and (TA@HA/Lys)_4_ coatings implanted for 4 (top) and 8 weeks (bottom). **(B)** Bone volume (BV) fraction [BV/total volume (TV)] of control, (TA@HA/Lys)_2_ and (TA@HA/Lys)_4_ coatings group. *p < 0.05, **p < 0.01, ***p < 0.001, ****p < 0.0001.

The osteogenic capacity of (TA@HA/Lys)_n_ coatings in animals was further evaluated by applying these coatings onto titanium rods, which were implanted at the distal end of the rabbit femur for 4 and 8 weeks. It was confirmed that all titanium rods had been accurately implanted in the rabbit femur. Micro-CT was used to detect new bone generation, and the CTVol software was used to establish a three-dimensional (3D) model of bone regeneration (Figure 10A). Both the control group and (TA@HA/Lys)_n_ coatings showed good bone regeneration ability at 4 and 8 weeks, with (TA@HA/Lys)_n_ coatings showing better findings than the control group (Figure 10A). After 4 weeks of implantation, there was no significant difference between (TA@HA/Lys)_2_ film and (TA@HA/Lys)_4_ film, while after 8 weeks of implantation, the amount of bone regeneration on (TA@HA/Lys)_4_ film was significantly increased in comparison with that of (TA@HA/Lys)_2_ film. The difference of bone regeneration between the control group and HA/Lys films was further extended at this time point (Figure 10B). The BV over tissue volume fractions (BV/TV) of (TA@HA/Lys)_2_ and (TA@HA/Lys)_4_ films were 1.3 and 1.5 times of that of the control group, respectively (Figure 10B). These results showed that (TA@HA/Lys)_n_ coating could promote new bone formation and accelerate bone healing. The reason for this may be that TA release reduces the production of ROS (Gülçin et al., 2010), and TA can promote adhesion between implants and cells and facilitate bone healing. The release of HA accelerates the differentiation of bone cells (Oonishi, 1991).

Infection and poor bone healing are common complications after the internal fixation of fractures. Once they occur, the failure rate increases (Schroer et al., 2013). Therefore, it is important to design a kind of implant material that can prevent or treat infection and poor bone healing. Titanium possesses excellent mechanical properties, low cytotoxicity, and good biocompatibility, but it can also make the implant surface bioinert, which is not conducive to bone healing (Qin et al., 2018). Therefore, the modification of the titanium surface is preferable. Antibacterial coating, such as with antibiotics, metal ions, and natural antibacterial materials, on the titanium surface is beneficial to inhibit bacterial adhesion and biofilm formation. Yuan et al. (2020) showed that a Lys/collagen nanofiber MATS have good antibacterial properties, mechanical properties, and biocompatibility. Lys possesses strong activity against *S. aureus* and *E. coli*. Shuai et al. (2021) showed that hydroxyapatite/poly-l-lactide (HAP/PLLA) scaffolds exhibit good ability to repair bone defects when HAP is released, and promote abundant new bone tissue formation. HA is widely used in bone implant materials and can be used to construct enhanced osteogenic coating on the surface of titanium. Studies (Yao et al., 2019) have shown that, when the bone is injured, a large number of free radicals will be produced at the site of injury while oxidative stress inhibits bone healing. In this context, antioxidant drugs can be administered to promote bone healing. Yang et al. (2019b) have shown that TA has the ability to reduce free radicals and promote bone healing.

Against this background, we believe that TA@HA/Lys films possess antioxidant properties. These films improve short-term cell adhesion and long-term cell proliferation, as well as promote osteogenic differentiation, and also enhance the generation of new bone *in vivo*. In addition, these films possess antibacterial properties. The incorporation of HA and Lys into a thin film in an LBL manner provides an alternative method for the application of the implantable coating in orthopedic materials. There is a clear need to develop multifunctional strategies that promote bone integration while reducing bacterial colonization (Raphel et al., 2016), as both outcomes are necessary to ensure the optimal long-term function of medical implants. In recent years, dual-function coating (Min et al., 2016), especially coating with antibacterial and osteogenic properties, has been extensively investigated, and accumulated evidence has shown that this improves osteointegration compared with mono-functional coating. However, osteointegration is a very complicated process, involving various types of cell, which influence the outcome by secreting different biomolecular signals. The promotion of bone formation and remodeling is a central issue in osteointegration, but could be disrupted by poor osteogenesis, infection, and stress due to excess ROS around implants, among others. Here, we proposed a multifunctional coating to overcome the challenge posed by the complexity of the process of osteointegration.

Hydroxyapatite in (TA@HA/Lys)_2_ plays a vital role in osteogenesis due to not only activating the osteogenic pathways by releasing Ca^2+^ and PO^3–^ but also facilitating the mineralization of extracellular matrix proteins and maturation of the extracellular matrix. Studies (Yao et al., 2019) have shown that a large number of free radicals are produced at the site of injury while oxidative stress inhibits bone healing and when the bone is injured, but bone healing is promoted when the body receives antioxidant drugs. The incorporated TA has a strong ability to scavenge ROS, which could regulate the ROS level around implants to ensure the proper function of various cells. In addition, the ability of TA to bind to metal ions, especially Ca ions, could contribute the osteogenesis as well. Compared with antibiotics, Lys, as an antibacterial protein widespread in animal and human tissues (Callewaert and Michiels, 2010), only kills the bacteria and is not harmful to normal tissue cells, so it could be widely used without a risk of abuse due to its specific antibacterial mechanism and low immune response. The LBL technique applied in this work was a very gentle process, which made the integrated gradients maintain their original biological activities as possible. In addition, its deposition in a manner independent of the shapes and materials of substrates could provide huge advantages for fabricating various implants. Integrating these components with different functions into the coating could be a promising option for producing multifunctional implants to meet the intensive demands on implants.

As reported, HA has favorable physical and chemical characteristics that foster osteogenesis *in vitro* and integration with surrounding tissue *in vivo* of animal trials (Shi et al., 2002). However, its effect on osteointegration was much smaller than previously expected, based on the outcomes of clinical trials in more than four decades (Surmenev and Surmeneva, 2019). From this point, the clinical trial should be carried out to further reveal the outcomes of such a composite coating on osteointegration. Topology of the coating, such as the micro- and nano-topography, is a significant aspect to enhance biofunctionalities, which is the shortcoming of the LBL technique applied in this work (Wang et al., 2019). In addition, the long-term stability of the coating is extremely important to the success in the clinical trial. Although the stability of this composite coating was tested, the time of 3 weeks was a much shorter period compared to the time in a clinical trial from several years to several 10 years.

## Conclusion

In this study, an inorganic/organic hybrid coating, (TA@HA/Lys)_n_, was constructed by integrating TA, HA, and Lys using the LBL technique, in which glue-like TA firmly stuck HA and Lys together. These coatings derived from the applied raw materials exhibited multiple functions, including antioxidant, sterilization, and promising osteogenesis effects, in both *in vitro* and *in vivo* experiments. In addition, upon the detection of MC3T3-E1 cells and DPSCs, (TA@HA/Lys)_n_ coatings were shown to accelerate the attachment in the early stage and proliferation in the long term. Importantly, (TA@HA/Lys)_n_ coatings greatly promoted Ca mineralization, osteogenesis, and bone formation *in vitro* and *in vivo*. Combining strong antioxidant and powerful antibacterial activities (sterilizing both *E. coli* and *S. aureus*), (TA@HA/Lys)_n_ was shown to be a promising candidate for coating onto orthopedic implants, to meet the challenges posed by the complexity of physiological conditions in the body and the intensive demands on orthopedic implants. Moreover, considering the multiple functionalities and abundant resources of polyphenols, the developed strategy of depositing polyphenols onto an inorganic material and subsequently incorporating this into an organic matrix could be beneficial for multifunctional hybrid materials, not only for orthopedic coatings, but also for additional applications in catalysts, sensors, tissue engineering, etc.

## Data Availability Statement

The original contributions presented in the study are included in the article/Supplementary Material, further inquiries can be directed to the corresponding author/s.

## Ethics Statement

All animal protocols were approved by the Ethics Committee of the Laboratory Animal Center of the Wenzhou Medical University, with approval license of experimental animals (SYXK 2015-009). All procedures were performed in accordance with the standard guidelines described in the Guide for the Care and Use of Laboratory Animals. Written informed consent was obtained from the owners for the participation of their animals in this study.

## Author Contributions

GW and YZ performed the conceptualization, methodology, experiments, data analysis, and writing. XZ and ML took part in the design of experiments, reviewed, and edited the draft. All authors read and approved the final manuscript.

## Conflict of Interest

The authors declare that the research was conducted in the absence of any commercial or financial relationships that could be construed as a potential conflict of interest. The reviewer JS declared a shared affiliation, with no collaboration, with the authors YZ and XZ to the handling editor at the time of the review.
