# Supplementary material for: Endowing Orthopedic Implants’ Antibacterial, Antioxidation, and Osteogenesis Properties Through a Composite Coating of Nano-Hydroxyapatite, Tannic Acid, and Lysozyme
Source: Front Bioeng Biotechnol. 2021 Jul 19;9:718255. doi: 10.3389/fbioe.2021.718255 (PMC8327088; doi:10.3389/fbioe.2021.718255)
Supplement: Supplementary file 1 [file Data_Sheet_1.DOCX]

Supplementary Material

# Supplementary Figures

**Figure S1.** The thickness of (TA /Lys)_6-pH_ constructed at different pHs.


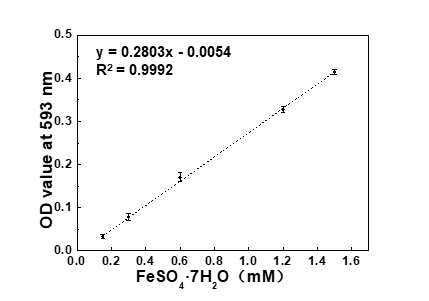


**Figure S2.** The standard curve obtained from reducing antioxidant power (FRAP) assay kit, tested at 593 nm with a spectrophotometer.
